# Glucose Depletion Enhances the Stem Cell Phenotype and Gemcitabine Resistance of Cholangiocarcinoma Organoids through AKT Phosphorylation and Reactive Oxygen Species

**DOI:** 10.3390/cancers11121993

**Published:** 2019-12-11

**Authors:** Nao Yoshikawa, Yoshimasa Saito, Hiroki Manabe, Toshiaki Nakaoka, Ryoei Uchida, Ryo Furukawa, Toshihide Muramatsu, Yuko Sugiyama, Masaki Kimura, Hidetsugu Saito

**Affiliations:** Division of Pharmacotherapeutics, Keio University Faculty of Pharmacy, 1-5-30 Shiba-kohen, Minato-ku, Tokyo 105-8512, Japan; na0marim0.6130ny@gmail.com (N.Y.); hirokitty.hellokitty@gmail.com (H.M.); toshiakinakaoka1116@gmail.com (T.N.); ryoei920403@gmail.com (R.U.); ryof0920@gmail.com (R.F.); ee.muramatsu.k@gmail.com (T.M.); kimura-ms@pha.keio.ac.jp (M.K.); saito-hd@pha.keio.ac.jp (H.S.)

**Keywords:** cholangiocarcinoma, glucose depletion, organoid culture, gemcitabine resistance, AKT phosphorylation, reactive oxygen species

## Abstract

Cancer cells are strongly dependent on the glycolytic pathway for generation of energy even under aerobic condition through a phenomenon known as the Warburg effect. Rapid proliferation of cancer cells is often accompanied by high glucose consumption and abnormal angiogenesis, which may lead to glucose depletion. In the present study, we investigated how cholangiocarcinoma cells adapt to glucose depletion using a 3D organoid culture system. We cultured organoids derived from cholangiocarcinoma under glucose-free condition and investigated cell proliferation, expression of stem cell markers and resistance to gemcitabine. Cholangiocarcinoma organoids cultured under glucose-free condition showed reduced proliferation but were able to survive. We also observed an increase in the expression of stem cell markers including *LGR5* and enhancement of stem cell phenotypic characteristics such as resistance to gemcitabine through AKT phosphorylation and reactive oxygen species. These findings indicate that cholangiocarcinoma cells are able to adapt to glucose depletion through enhancement of their stem cell phenotype in response to changes in microenvironmental conditions.

## 1. Introduction

Most cancer cells rely predominantly on anaerobic glycolysis to generate energy even under aerobic condition, a phenomenon known as the “Warburg effect” [1,2]. Since glycolysis yields only two molecules of ATP, cancer cells require a large amount of glucose for energy production. In fact, it has been shown that the glucose concentration in colonic or gastric cancer tissue is only one tenth of that in normal tissue, whereas the concentration of lactate, the final product of glycolysis, is significantly elevated [3]. However, rapid proliferation of cancer cells results in abnormal angiogenesis, which means that cancer tissues often become depleted of oxygen and nutrients, including glucose [4]. We have recently demonstrated that liver cancer cells can survive even under hypoglycemic condition through microRNA-mediated gene regulation [5].

Cholangiocarcinoma is a highly malignant adenocarcinoma originating from bile duct epithelial cells. The incidence of cholangiocarcinoma is apparently increasing worldwide. Although surgical resection is the only curative treatment for affected patients, most cases are diagnosed at an advanced stage and have poor outcomes [6]. Patients with inoperable cholangiocarcinoma generally receive chemotherapy regimens including gemcitabine, but their effects are limited, and the 5-year survival rates are still low [7,8,9]. 

Cancer stem cells represent a subpopulation of cancer cells exhibiting self-renewal ability and pluripotency, being integral to tumor development, recurrence and drug resistance. Organoid culture is a 3D culture system that allows long-term expansion of LGR5-positive stem cells into cyst-like structures (organoids) with properties resembling those of the original tissues. LGR5, a member of the Wnt signaling pathway, has been identified as a new molecular marker of stem cells in the small intestine, colon, stomach, liver and pancreas. This type of 3D culture system involves the use of serum-free medium that includes only defined factors such as R-spondin 1 (Rspo1), epidermal growth factor (EGF) and Noggin, and has allowed the establishment of organoid models of intestinal, prostatic, pancreatic and liver tumors [10,11,12,13,14,15]. We have recently developed several organoids derived from intrahepatic cholangiocarcinoma (ICC) [16]. In the present study, to investigate the molecular mechanism by which cancer cells adapt to extracellular glucose depletion, we cultured organoids derived from human ICC under glucose-free condition and examined their growth activity, stem cell phenotype and resistance to gemcitabine. 

## 2. Materials and Methods

### 2.1. Organoid Culture and Glucose Conditions

Two human cholangiocarcinoma organoid lines (termed CCO1 and CCO2) were established from ICC tissues obtained from patients and stably cultured as described previously [16]. In the present study, we cultured CCO1 and CCO2 under two different glucose conditions. 

The glucose-containing medium (Glu (+)) was Advanced DMEM/F12 (Thermo Fisher Scientific, Waltham, MA, USA), and the glucose-free medium (Glu (−)) was SILAC Advanced DMEM/F12 Flex Media, without glucose or phenol red (Thermo Fisher Scientific). These basal media were supplemented with 1× Glutamax, 10 mM HEPES, 1× penicillin/streptomycin, 1× N2 supplement, 1× B27 supplement, 50 ng/mL EGF (all from Thermo Fisher Scientific), 10 nM gastrin, 10 mM nicotinamide (all from Sigma-Aldrich, St. Louis, MO, USA) and Rspo1 (10% conditioned medium from Rspo1-producing cell lines).

### 2.2. Drugs

The drugs employed were LY294002 (Cell Signaling Technology, Danvers, MA, USA) as an inhibitor of phosphatidylinositol 3-kinase (PI3K)-dependent AKT phosphorylation, N-acetylcysteine (NAC, Sigma-Aldrich), Tempol (Santa Cruz Biotechnology, Dallas, TX, USA), Cisplatin (AdipoGen, San Diego, CA, USA) and gemcitabine hydrochloride (Sigma-Aldrich). Final drug concentrations employed were 1.4 µM for LY294002, 1.25 mM for NAC, 1.25 mM for Tempol, 10 nM for Cisplatin and 10 nM for gemcitabine hydrochloride. 

### 2.3. Western Blotting

Western blotting was performed according to the method described previously [17]. In brief, protein extracts (10 µg) were separated by SDS/polyacrylamide gel electrophoresis and transferred to nitrocellulose membranes. The membranes were then hybridized with antibodies against AKT (Cell Signaling Technology 9272; 1/500) and phospho-AKT (Ser473, Cell Signaling Technology 9271, 1/500; Thr308, Cell Signaling Technology 9275, 1/500). Immunoreactivity was detected with ECL Select Western Blotting Detection Reagents (GE Healthcare Life Science, Marlborough, MA, USA) and imaged by Image Quant LAS 500 (GE Healthcare Life Science). An antibody against β-actin (C4) (Santa Cruz Biotechnology sc-47778; 1/5000) was used as an internal control. The band signal obtained was quantified using Image J and the ratio of p-AKT to β-actin expression was calculated. The whole images of Western blotting are shown in Supplementary Figure S1.

### 2.4. Quantitative RT-PCR

RNA extraction and quantitative RT-PCR were performed as described previously [5]. In brief, total RNAs were extracted from organoids using the RNeasy Mini kit (Qiagen, Hilden, Germany) and cDNAs were synthesized using Multiscribe Reverse Transcriptase (Thermo Fisher Scientific). Quantitative RT-PCR was performed using the Universal SYBR Select Master Mix (Thermo Fisher Scientific) in accordance with the manufacturer’s instructions. Quantitative analyses were performed using the CFX96 Real-Time System (BioRad, Hercules, CA, USA). GenBank accession number, primer sequence and melting temperature for each gene analyzed by quantitative RT-PCR are shown in Supplementary Table S1. *GAPDH* was used as an internal control. All experiments were carried out in triplicate. 

### 2.5. Flow Cytometry

Flow cytometry was performed in accordance with a previous study [18]. For counting of LGR5-positive cells, purified cells were fixed with 4% paraformaldehyde for 10 min and then treated with 0.1% PBS-Tween for 20 min and incubated with 10% normal donkey serum in PBS for 1 h at room temperature. This was followed by mixing with anti-LGR5 antibody (Abcam, Cambridge, UK; ab75732) at 4 °C with rotation. Isotype IgG (Abcam; ab37415) was also used. After washing with PBS, the cells were reacted with goat anti-rabbit IgG H&L (Alexa Fluor^®^ 488; Abcam; ab150077d) resuspended in 1 mL of PBS, and flow cytometry was performed with a BD LSR Ⅱ Flow Cytometer (BD Biosciences, San Jose, CA, USA). For measurement of intracellular H_2_O_2_, purified cells were suspended in BES-H_2_O_2_-Ac (Wako) at 1 µM for 1 h and flow cytometry was performed with a BD LSR Ⅱ Flow Cytometer. For apoptosis assay, purified cells were suspended in 500 µL 1× binding buffer, and propidium iodide was added at 50 µg/mL in the dark at room temperature for 5 min. Flow cytometry was performed with a BD LSR Ⅱ Flow Cytometer.

### 2.6. Measurement of ROS and Antioxidant Capacity

Cells from CCO1 and CCO2 were ultrasonicated and Reactive Oxygen Species (ROS) and antioxidant capacity were measured. Levels of ROS and antioxidant capacity were measured by diacron-reactive oxygen metabolites (d-ROMs) test and biological antioxidant potential (BAP) test using FREE carpe diem (Wismerll, Tokyo, Japan) according to the manufacturer’s instructions.

### 2.7. Statistics

Data were analyzed using the SPSS statistical software package. Student’s *t* test and Dunnett’ s test were applied for this study. Differences at *p* < 0.05 were considered significant.

## 3. Results

### 3.1. Glucose-Free Condition Reduce the Growth Activity of Cholangiocarcinoma Organoids

**Figure 1:**
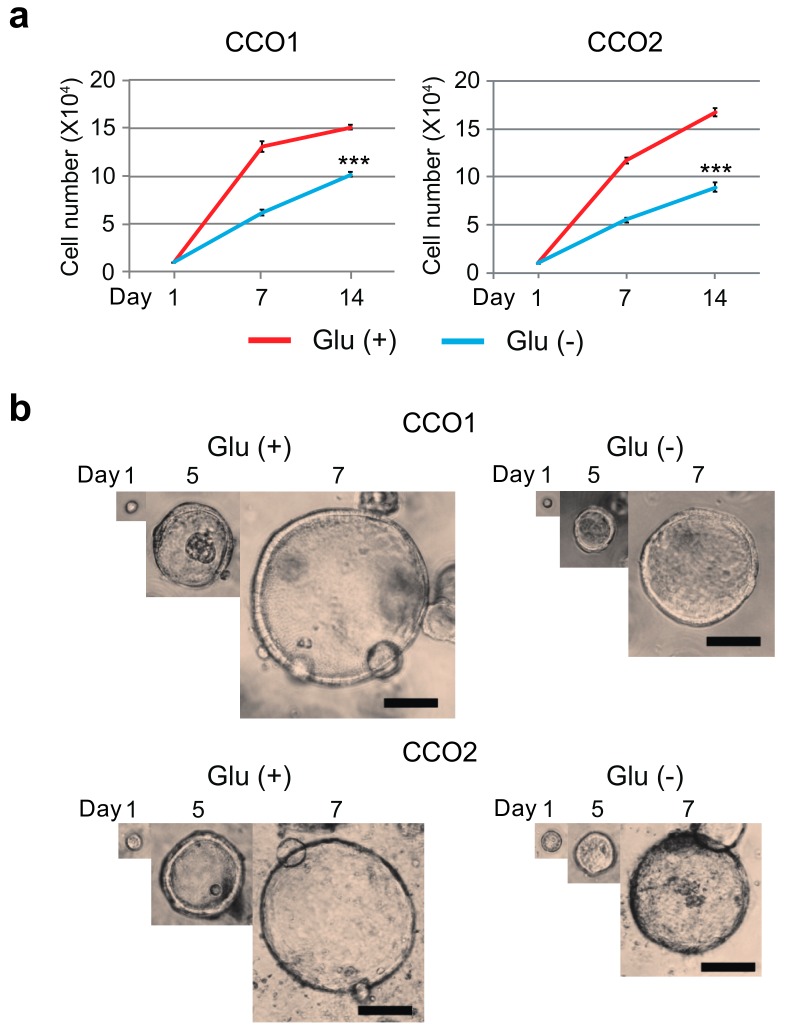
Growth activity of cholangiocarcinoma organoids cultured with or without glucose. (**a**) Growth activities of CCO1 and CCO2 cultured with or without glucose were evaluated by cell counting for 14 days. *** *p* < 0.001. (**b**) Bright-field images of CCO1 and CCO2 showing their size in culture medium with or without glucose. Scale bar: 200 µm.

The human cholangiocarcinoma organoid lines CCO1 and CCO2 were cultured under two different conditions Glu (+) or Glu (−) for 14 days, and their growth activities were evaluated by counting viable cells. The numbers of viable cells among CCO1 and CCO2 cultured under Glu (−) condition were significantly reduced in comparison to cells cultured under Glu (+) condition (Figure 1a). In addition, as shown in Figure 1b, the size of each organoid cultured under Glu (−) condition was smaller than that cultured under Glu (+) condition in CCO1 and CCO2. 

### 3.2. Higher Expression of Stem Cell Markers in Cholangiocarcinoma Organoids Cultured under Glucose-Free Condition

**Figure 2:**
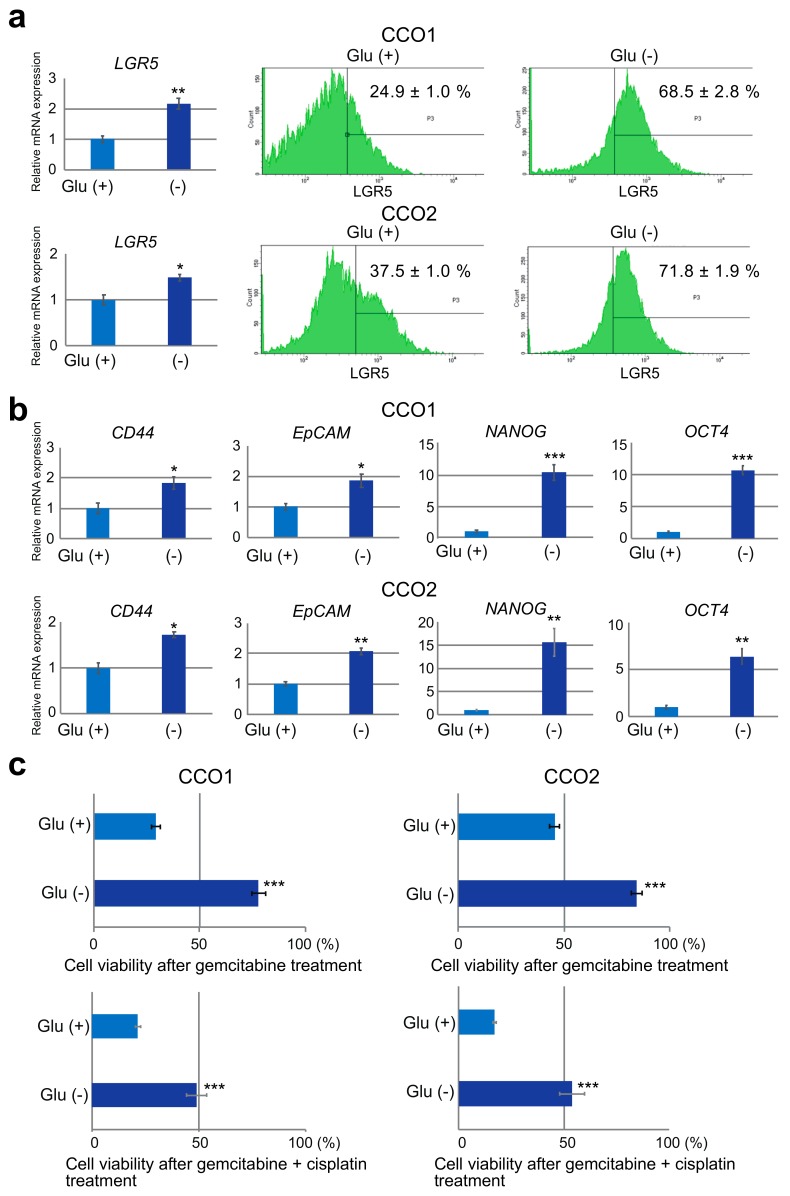
Expression of stem cell markers and sensitivity to gemcitabine in cholangiocarcinoma organoids cultured with or without glucose. (**a**) Expression levels of *LGR5* determined by quantitative RT-PCR and flow cytometric analysis in CCO1 and CCO2 cultured with or without glucose. ** *p* < 0.01. (**b**) Expression levels of the stem cell markers (*CD44, EpCAM, NANOG, OCT4*) in CCO1 and CCO2 cultured with or without glucose. * *p* < 0.05, ** *p* < 0.01, *** *p* < 0.001. (**c**) Cell viability after 7 days of treatment with gemcitabine and combination treatment with gemcitabine and cisplatin in CCO1 and CCO2 cultured with or without glucose. The percentages represent the ratio of gemcitabine-treated cells relative to non-treated cells. *** *p* < 0.001.

Although cholangiocarcinoma organoids cultured under Glu (−) condition were small and showed lower proliferative activity, as shown in Figure 1, they remained viable and continued to proliferate slowly, suggesting that cholangiocarcinoma organoids were able to survive even under the stress of glucose depletion. Therefore, we investigated alterations in stemness under different glucose conditions. The levels of expression of the stemness markers *LGR5*, *CD44*, *EpCAM*, *NANOG* and *OCT4* were examined by quantitative RT-PCR and compared between Glu (+) and Glu (−) conditions. As shown in Figure 2a,b, the expression of all stem cell markers was significantly increased in CCO1 and CCO2 under Glu (−) condition. In addition, we counted LGR5-positive cells using flow cytometry, and found that they were significantly increased in CCO1 and CCO2 under Glu (−) condition (Figure 2a). These results indicated that the stemness of cholangiocarcinoma organoids was increased in glucose-depleted culture.

### 3.3. Reduction of Gemcitabine Sensitivity in Cholangiocarcinoma Organoids under Glucose-Free Condition

The survival of cholangiocarcinoma organoids exhibiting a stronger stem cell phenotype under glucose-deficient condition suggested that this cell type was possibly resistant to anti-cancer agents. Since the first choice of chemotherapy for cholangiocarcinoma is a regimen including gemcitabine, we investigated the sensitivity of cholangiocarcinoma organoids to gemcitabine in the presence or absence of glucose for 7 days. As shown in Figure 2c, cell viability after gemcitabine treatment indicated that CCO1 and CCO2 were sensitive to gemcitabine after culture under Glu (+) condition. On the other hand, CCO1 and CCO2 became resistant to gemcitabine after culture under Glu (−) condition. We also treated cholangiocarcinoma organoids with combination of gemcitabine and cisplatin. The cell viabilities of CCO1 and CCO2 after combination treatment with gemcitabine and cisplatin were significantly increased under Glu (−) condition compared to Glu (+) condition (Figure 2c). These findings suggest that sensitivity to gemcitabine was reduced in cholangiocarcinoma organoids cultured under glucose-free condition.

### 3.4. Increase of AKT Phosphorylation in Cholangiocarcinoma Organoids Cultured under Glucose-Free Condition

**Figure 3:**
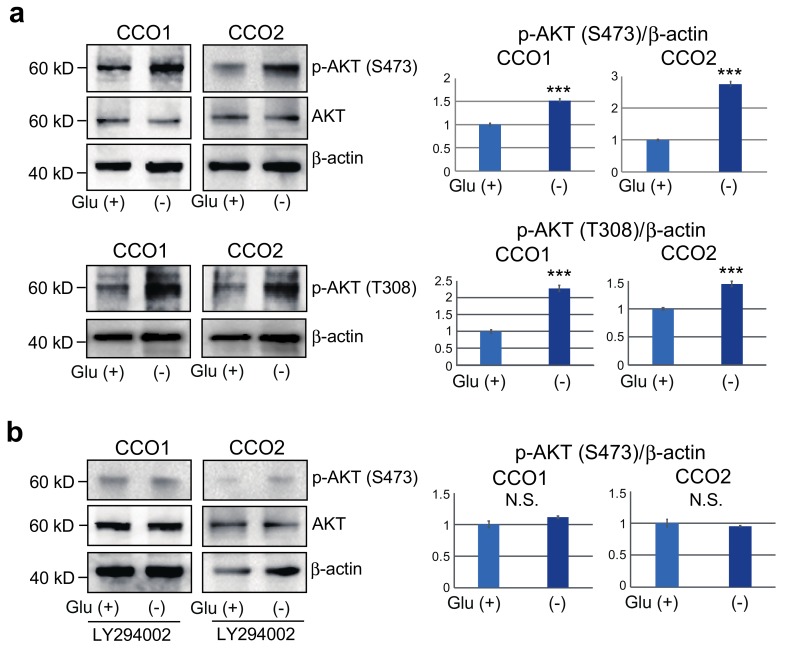
AKT phosphorylation in cholangiocarcinoma organoids cultured with or without glucose. (**a**) Western blotting of phosphorylated AKT (p-AKT S473 and T308) in CCO1 and CCO2 cultured with or without glucose. Graphs in the right panel show the ratio of signal intensities of p-AKT relative to β-actin. *** *p* < 0.001. (**b**) Western blotting of p-AKT in CCO1 and CCO2 cultured with or without glucose after treatment with the inhibitor of AKT phosphorylation, LY294002. N.S.: not significant. The whole images of Western blotting are shown in Supplementary Figure S1.

It has been reported that glucose depletion induces hyperphosphorylation of AKT, which is closely related to tumor survival and enlargement in several cancer cell lines [19,20,21]. Accordingly, we investigated AKT phosphorylation (Ser473 and Thr308) after culture of cholangiocarcinoma organoids for 7 days under two different glucose conditions. Levels of AKT phosphorylation of both Ser473 and Thr308 were weak in CCO1 and CCO2 when cultured under Glu (+) condition, but significantly increased under Glu (−) condition (Figure 3a). We then treated CCO1 and CCO2 with LY294002, the inhibitor of PI3K-dependent AKT phosphorylation. As shown in Figure 3b, there was no significant difference in the expression of phosphorylated AKT between Glu (+) and Glu (−) conditions in CCO1 and CCO2 after LY294002 treatment. These findings indicated that AKT phosphorylation is increased in cholangiocarcinoma organoids cultured under glucose-free condition.

### 3.5. Inhibition of AKT Phosphorylation under Glucose-Free Condition Leads to A Decrease in Stemness and Resistance to Gemcitabine in Cholangiocarcinoma Organoids

**Figure 4:**
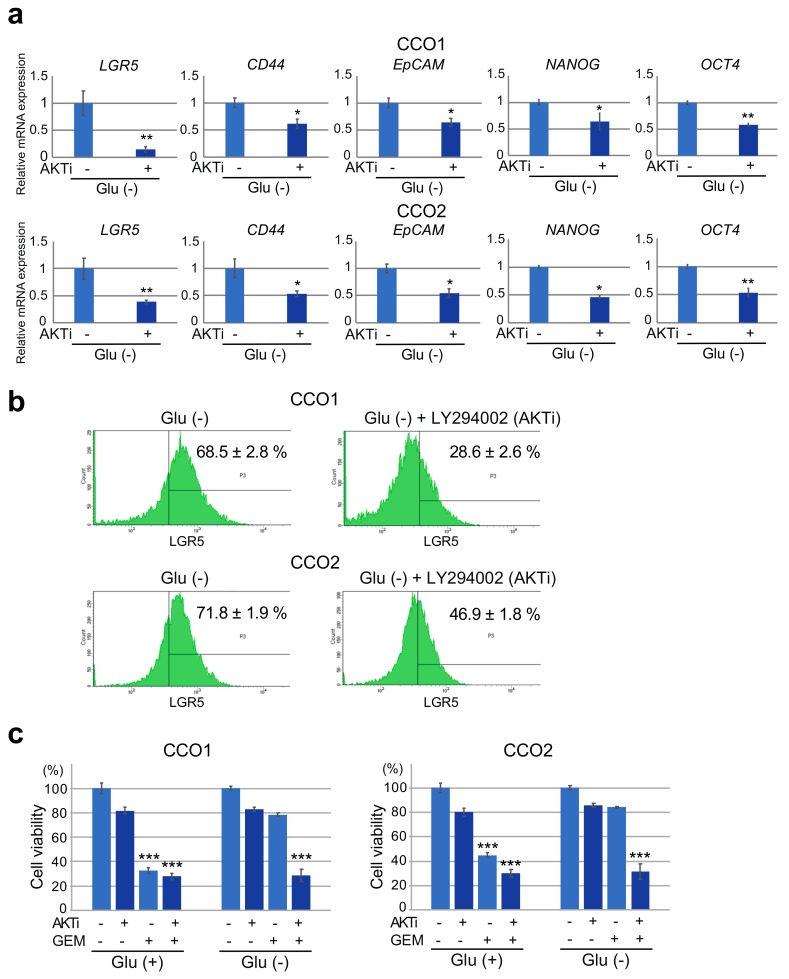
Expression of stem cell markers and sensitivity to gemcitabine in cholangiocarcinoma organoids cultured with or without glucose after treatment with the AKT phosphorylation inhibitor. (**a**) Expression of stem cell markers (*LGR5, CD44, EpCAM, NANOG, OCT4*) in CCO1 and CCO2 cultured without glucose after treatment with the AKT phosphorylation inhibitor (AKTi), LY264002. * *p* < 0.05, ** *p* < 0.01. (**b**) Flow cytometric analysis of LGR5-positive cells in CCO1 and CCO2 cultured with or without glucose after 7 days of treatment with LY294002. (**c**) Cell viability after 7 days of treatment with LY294002 (AKTi) and/or gemcitabine (GEM) in CCO1 and CCO2 cultured with or without glucose. The percentages represent the ratio of cell viability in cells treated with each drug relative to non-treated cells. *** *p* < 0.001.

We next examined the effect of inhibition of AKT phosphorylation on stemness in cholangiocarcinoma organoids under hypoglycemic condition. After culture under Glu (−) condition for 7 days, CCO1 and CCO2 were treated with LY294002. As shown in Figure 4a, expression levels of stem cell markers *LGR5*, *CD44*, *EpCAM*, *NANOG* and *OCT4* were significantly reduced in CCO1 and CCO2 cultured under Glu (−) condition when AKT phosphorylation was inhibited by LY294002. Flow cytometric analysis also demonstrated that the number of LGR5-positive cells was significantly decreased in CCO1 and CCO2 cultured under Glu (−) condition when AKT phosphorylation was inhibited by LY294002 (Figure 4b). 

As shown in Figure 4c, alterations in the numbers of viable cells in CCO1 and CCO2 after exposure to LY294002 and gemcitabine differed between Glu (+) and Glu (−) culture conditions. Under Glu (+) condition, the number of viable cells in CCO1 and CCO2 was decreased by gemcitabine treatment, irrespective of exposure to LY294002. On the other hand, under Glu (−) condition, the number of viable cells among CCO1 and CCO2 was not decreased by gemcitabine treatment in the presence of LY294002 (Figure 4c). The number of viable cells in CCO1 and CCO2 was decreased only after treatment with both gemcitabine and LY294002 under Glu (−) condition, suggesting that the resistance of cholangiocarcinoma organoids to gemcitabine under glucose-free condition was dependent on AKT phosphorylation (Figure 4c). These results indicate that inhibition of AKT phosphorylation under glucose-free condition led to a decrease in stemness and resistance to gemcitabine in cholangiocarcinoma organoids. 

### 3.6. Stemness and Gemcitabine Resistance of Cholangiocarcinoma Organoids Cultured under Glucose-Free Condition are Mediated by Reactive Oxygen Species (ROS) 

ROS are known to play an important role in activation of the AKT pathway in cancer cells. Under hypoxic condition, an increase of ROS induces AKT activation, and the downstream molecules in the AKT signaling pathway stimulate proliferation and invasion of cancer cells [22,23]. Our results also suggested that AKT phosphorylation was activated under glucose-free condition, indicating the involvement of ROS in the stemness of cholangiocarcinoma organoids. 

**Figure 5:**
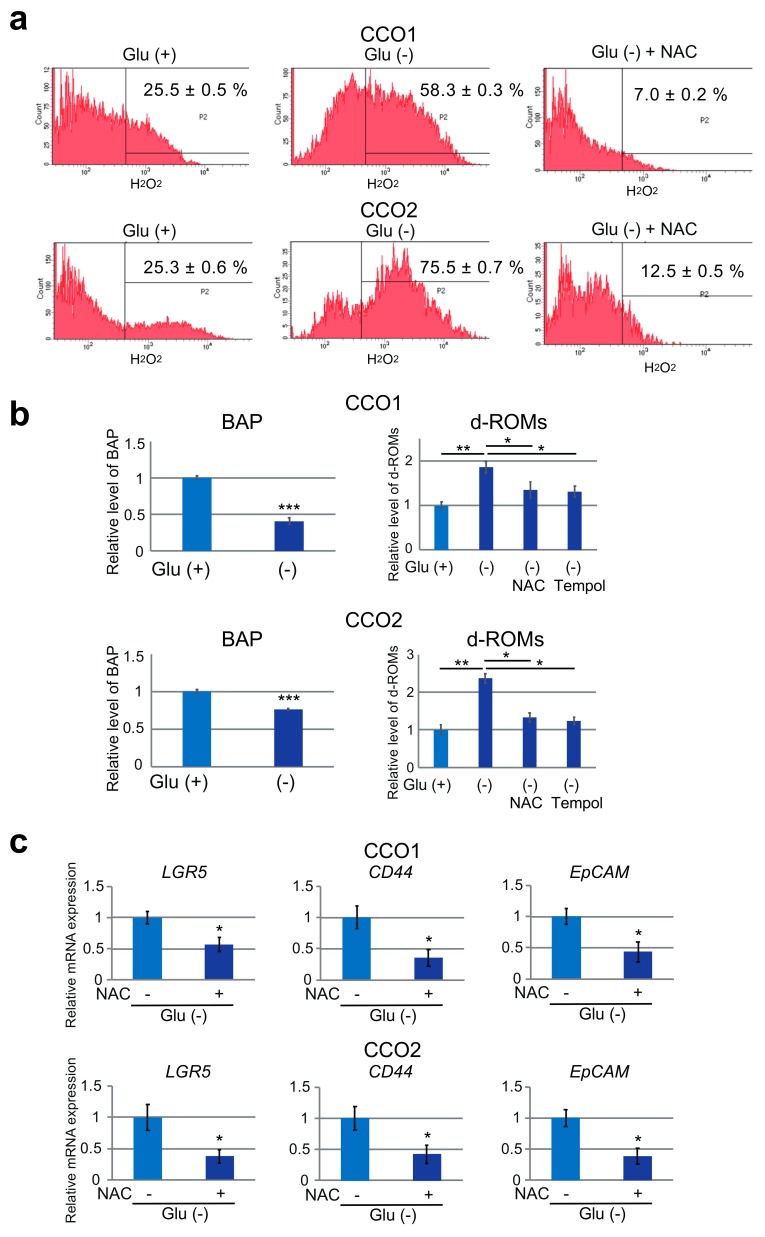
Involvement of ROS in the stem cell phenotype of cholangiocarcinoma organoids cultured with or without glucose. (**a**) Flow cytometric analysis of intracellular H_2_O_2_ in CCO1 and CCO2 cultured with or without glucose as well as glucose-free condition after treatment with the ROS inhibitor, NAC. (**b**) Biological antioxidant potential (BAP) test and diacron-reactive oxygen metabolites (d-ROMs) test to estimate antioxidant capacity and ROS in CCO1 and CCO2 cultured with or without glucose as well as glucose-free condition after treatment with antioxidants NAC and Tempol. * *p* < 0.05, ** *p* < 0.01, *** *p* < 0.001. (**c**) Expression of stem cell markers (*LGR5, CD44, EpCAM*) in CCO1 and CCO2 cultured with or without glucose after treatment with NAC. * *p* < 0.05.

Using flow cytometry, we measured the level of intracellular H_2_O_2_ in CCO1 and CCO2 after 7 days of culture under Glu (+) or Glu (−) condition. As shown in Figure 5a, the level of H_2_O_2_ was significantly elevated in CCO1 and CCO2 cultured under glucose-free condition. The antioxidant NAC significantly reduced H_2_O_2_ in CCO1 and CCO2 under glucose-free condition (Figure 5a). In addition, we performed BAP test and d-ROMs test to estimate antioxidant capacity and ROS in cholangiocarcinoma organoids, respectively. As shown in Figure 5b, the levels of antioxidant capacity were significantly decreased and the levels of ROS were significantly increased in CCO1 and CCO2 cultured under glucose-free condition. 

We also employed Tempol as an alternative antioxidant and validated that the levels of ROS were significantly decreased by Tempol as well as NAC in CCO1 and CCO2 cultured under glucose-free condition (Figure 5b). We then examined the expression of *LGR5*, *CD44* and *EpCAM* in CCO1 and CCO2 cultured under glucose-free condition with or without NAC, and found that it was significantly reduced by NAC treatment (Figure 5c).

**Figure 6:**
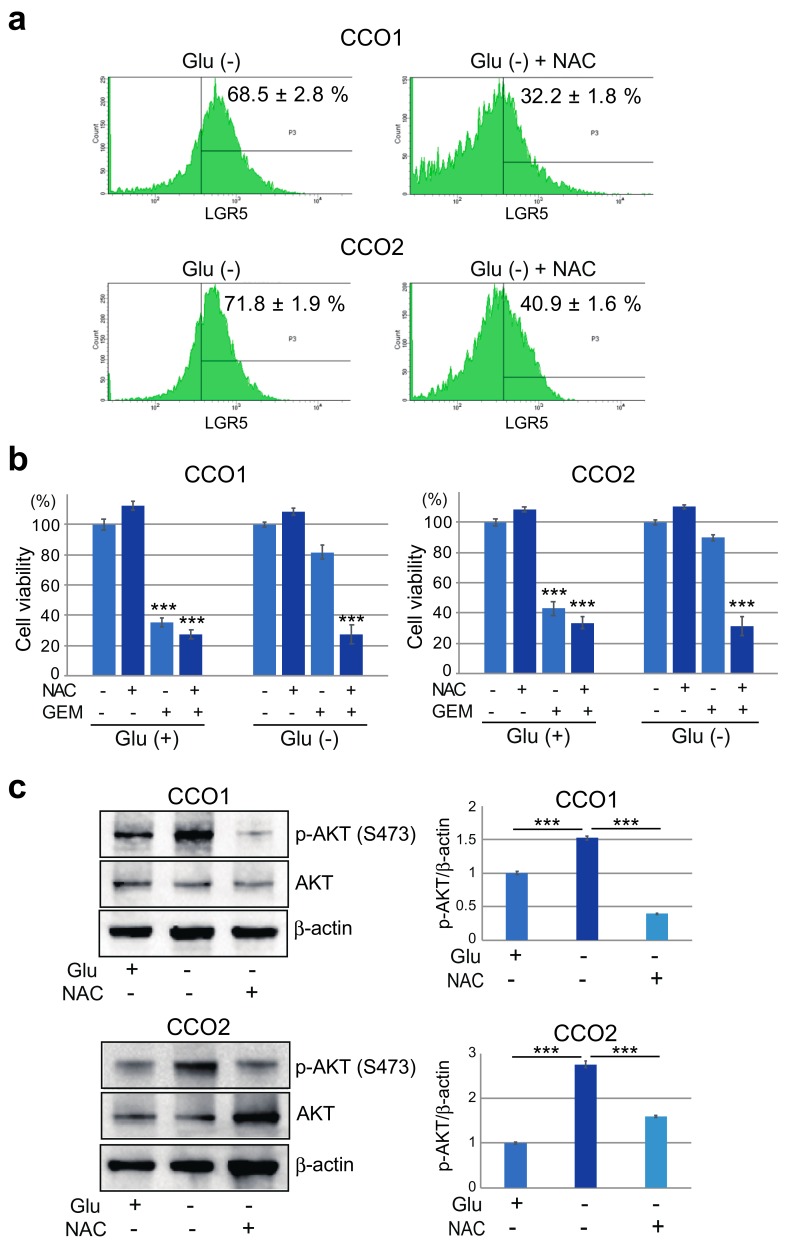
Effect of NAC on LGR5 expression, gemcitabine resistance and AKT phosphorylation. (**a**) Flow cytometric analysis of LGR5-positive cells in CCO1 and CCO2 cultured with or without glucose after treatment with NAC. (**b**) Cell viabilities after 7 days treatment with NAC and/or gemcitabine (GEM) in CCO1 and CCO2 cultured with or without glucose. The percentages represent the ratio of cell viability in cells treated with each drug relative to non-treated cells. *** *p* < 0.001. (**c**) Western blotting of p-AKT in CCO1 and CCO2 cultured with or without glucose after 7 days treatment with NAC. Graphs in the right panel show the ratio of signal intensity of p-AKT relative to that of β-actin. *** *p* < 0.001. The whole images of Western blotting are shown in Supplementary Figure S1.

Flow cytometric analysis also demonstrated that the percentage of LGR5-positive cells was significantly reduced by NAC treatment in CCO1 and CCO2 (Figure 6a), suggesting that suppression of ROS reduced the number of cholangiocarcinoma organoids with a stem cell phenotype. We then examined the effect of NAC on gemcitabine sensitivity in CCO1 and CCO2 cultured under Glu (+) or Glu (−) condition. Alterations in the number of viable cells in CCO1 and CCO2 after exposure to NAC and gemcitabine differed between the two glucose conditions (Figure 6b). Under Glu (+) condition, the number of viable cells in CCO1 and CCO2 was decreased by gemcitabine treatment, irrespective of the presence of NAC. On the other hand, under Glu (−) condition, the number of viable cells was not decreased by gemcitabine in the absence of NAC treatment, but was decreased after treatment with both gemcitabine and NAC (Figure 6b). Moreover, the increased AKT phosphorylation observed in CCO1 and CCO2 under glucose-free condition was inhibited by NAC treatment (Figure 6c). These observations suggest that the stemness and gemcitabine resistance of cholangiocarcinoma organoids under glucose-free condition are mediated by ROS.

### 3.7. AKT Phosphorylation and ROS Increase the Stemness of Cholangiocarcinoma Organoids Transferred from Glucose-Sufficient to Glucose-Free Condition

Our results demonstrated that cholangiocarcinoma organoids were able to survive even under glucose-free culture condition and exhibit a stem cell-like phenotype. Cancer cells are exposed to various concentrations of glucose depending on host nutritional status and the state of angiogenesis in cancer tissues. Not only cancer stem cells but also differentiated cancer cells without a stem cell-like phenotype are exposed to the same glucose-free condition. Therefore, to investigate the response of cholangiocarcinoma organoids after transfer from glucose-sufficient to glucose-free condition, we analyzed stemness of organoids that had been cultured with glucose for 7 days, followed by 7 days of culture under either Glu (+) or Glu (−) condition, i.e., Glu (+ → +) vs. Glu (+ → −). 

**Figure 7:**
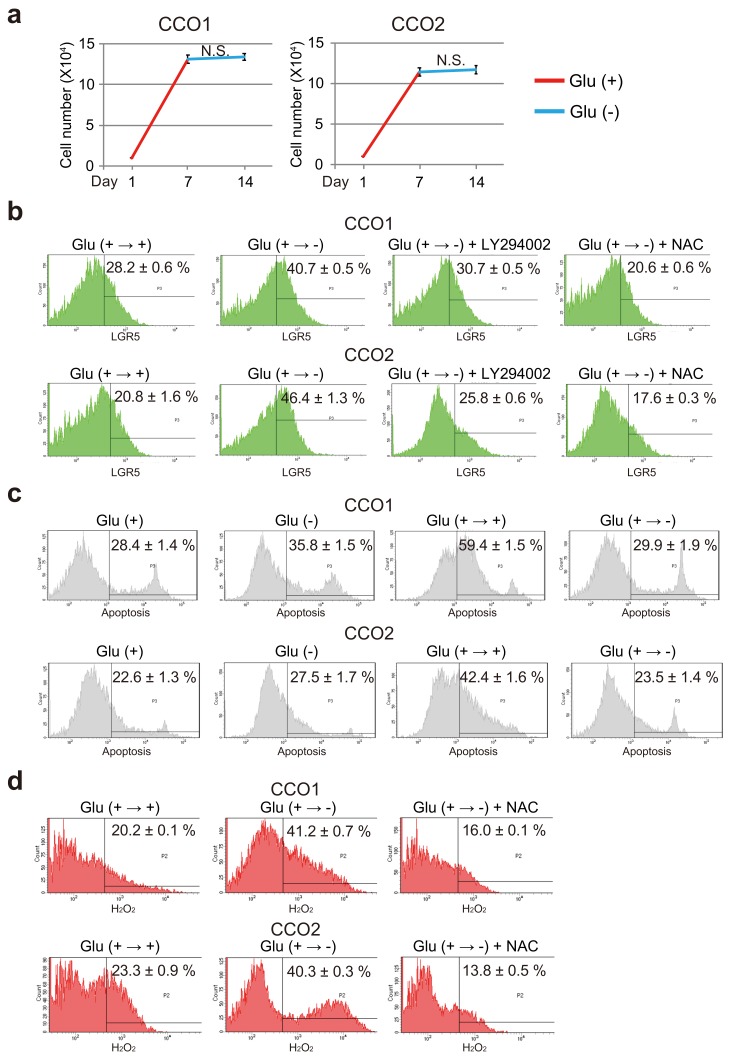
Stem cell phenotype in cholangiocarcinoma organoids cultured under glucose-free condition after transfer from glucose-sufficient condition. (**a**) Growth activity of CCO1 and CCO2 exposed to glucose for 7 days followed by exposure to glucose-free condition for 7 days. N.S.; not significant. (**b**) Flow cytometric analysis of LGR5-positive cells in CCO1 and CCO2 exposed to glucose for 7 days followed by exposure to glucose-free condition for 7 days (i.e., Glu (+ → −)) in addition to treatment with LY294002 and NAC.(**c**) Flow cytometric analysis of apoptotic cells in CCO1 and CCO2 exposed to glucose for 7 days followed by culture with or without glucose for 7 days (i.e., Glu (+ → +) or Glu (+ → −)). (**d**) Flow cytometric analysis of intracellular H_2_O_2_ in CCO1 and CCO2 exposed to glucose for 7 days followed by exposure to glucose-free condition for 7 days (i.e., Glu (+ → −)) in addition to treatment with NAC.

As shown in Figure 7a, the number of viable cells among CCO1 and CCO2 increased during Glu (+) culture (days 0–7), but did not increase during Glu (−) culture (days 7–14). Flow cytometry showed that the number of LGR5-positive cells was significantly increased under Glu (+ → −) condition relative to Glu (+ → +) condition in CCO1 and CCO2 (Figure 7b). 

To investigate whether cancer stem cells were increased or differentiated cancer cells obtained stemness in cholangiocarcinoma organoids cultured under glucose-free condition, we analyzed late apoptotic cells by PI staining. The number of apoptotic cells under Glu (+ → −) condition was almost the same level as Glu (+) condition, whereas Glu (+ → +) condition showed a significant increase of apoptotic cells in CCO1 and CCO2 (Figure 7c). This suggests that culture under glucose-free condition may lead to dedifferentiation of differentiated cancer cells to cancer stem cells in cholangiocarcinoma organoids.

We also confirmed that LY294002, the inhibitor of AKT phosphorylation, decreased the number of LGR5-positive cells in CCO1 and CCO2 cultured under Glu (+ → −) condition (Figure 7b). NAC treatment also reduced the number of LGR5-positive cells in CCO1 and CCO2 cultured under Glu (+ → −) condition (Figure 7b). Examination of the level of intracellular H_2_O_2_ in CCO1 and CCO2 cultured under Glu (+ → +) and Glu (+ → −) condition showed that H_2_O_2_ was significantly increased during glucose-free culture, and that NAC, a precursor of glutathione, reduced the amount of H_2_O_2_ (Figure 7d). These results indicated that AKT phosphorylation and ROS increased the stemness of cholangiocarcinoma organoids that had been initially cultured under glucose-sufficient condition and then transferred to glucose-free condition.

## 4. Discussion

Cancer cells are strongly dependent on the glycolytic pathway for generation of energy even under aerobic condition, a phenomenon known as the Warburg effect. In addition, cancer cells are exposed to various glucose concentrations resulting from changes in microenvironmental conditions such as host nutritional status and angiogenesis in cancer tissues. Rapid proliferation of cancer cells often results in increased consumption of glucose and abnormal angiogenesis, which may lead to glucose depletion. However, the physiology of cancer cells including cancer stem cells under glucose-free condition remains unclear. Here, using a 3D organoid culture system, we investigated how cholangiocarcinoma cells adapt to glucose depletion. We demonstrated that cholangiocarcinoma organoids were able to survive even under glucose-free condition by increasing their cell stemness, consequently acquiring resistance to gemcitabine. We also showed that AKT phosphorylation and ROS played a critical role in this phenotypic change. 

It has been reported that some cellular stressors such as hypoxia lead to an increase of cancer stem cells in tumors and induce a drug-resistant phenotype [4]. Here we also found that glucose depletion in cholangiocarcinoma organoids induced an increase in their expression of stem cell markers, including *LGR5*, *CD44*, *EpCAM*, *NANOG* and *OCT4* and more pronounced stem cell phenotypic characteristics such as resistance to gemcitabine. These results suggested that cancer stem cells were able to self-proliferate even under glucose-free condition, whereas differentiated cancer cells became apoptotic. Therefore, the acquisition of resistance to gemcitabine in cholangiocarcinoma organoids under glucose-free condition was probably attributable to an increase in the population of cancer stem cells. 

We also found that cell survival and drug resistance in cholangiocarcinoma organoids under glucose-free condition occurred as a result of AKT phosphorylation. It has been reported that glucose depletion induces AKT phosphorylation [19,20,21] and that the AKT pathway plays an important role in resistance to cancer therapy [24,25,26,27,28]. Here, phosphorylation of AKT was elevated in cholangiocarcinoma organoids under glucose-free condition, and inhibition of AKT phosphorylation by LY294002 led to a decrease of stemness and drug resistance, suggesting that enhancement of the stem cell phenotype in cancer cells under condition of glucose depletion requires AKT phosphorylation. We examined expression levels of genes associated with the AKT signaling such as *mTOR* and the *BCL2* superfamily in cholangiocarcinoma organoids under glucose-free condition after treatment with LY294002, but there was no significant difference in the expression levels of these genes by inhibition of AKT phosphorylation (Supplementary Figure S2).

ROS are produced by mitochondria using NADPH oxidase (NOX) or nitric oxide synthase (NOS) and converted to H_2_O_2_ by superoxide dismutase (SOD), followed by further breakdown to H_2_O by antioxidants such as catalase and peroxidase [29]. Intracellular metabolic stresses sometimes induce ROS, which has been reported to exert both stimulatory and inhibitory effects on malignant tumors and induce upregulation of AKT expression [29,30,31,32,33]. H_2_O_2_ is known to be a more weakly reactive molecule than other intracellular ROS molecules, and is also a candidate intracellular signaling molecule [34,35,36]. Osawa et al. have demonstrated that NOX4 produces H_2_O_2_ and activate AKT phosphorylation by glucose depletion in liver cancer cells [19]. It has also been reported that AKT activation increases the expression of NOX family proteins [37,38]. Exposure of cholangiocarcinoma organoids to glucose-depleted condition increased the intracellular concentration of H_2_O_2_ and the expression of LGR5. These increases were inhibited by treatment with the ROS inhibitor, NAC, suggesting that ROS play an important role in enhancement of the stem cell phenotype. Thus, the stem cell phenotype of cholangiocarcinoma organoids under glucose-depleted condition appears to require a certain amount of H_2_O_2_ and subsequent AKT phosphorylation. We also examined the effect of glucose depletion on differentiated cancer cells by culturing organoids in the presence of glucose during a period in which differentiated cancer cells were considered to increase. In the following glucose-free phase, the total number of cells did not change, but the number of LGR5-positive cells was increased, suggesting that the population of cancer stem cells was increased by transfer of organoids from glucose-sufficient to glucose-depleted condition. 

## 5. Conclusions

Our study of cholangiocarcinoma cells using a 3D organoid culture system has demonstrated that glucose-depleted condition lead to an increase of stem cell phenotypic characteristics such as resistance to gemcitabine through AKT phosphorylation and ROS. Cancer cells are usually dependent on the glycolytic pathway for energy. But even if cancer cells are exposed to glucose depletion due to changes in microenvironmental conditions, they are able to survive by enhancing their stemness. Inhibition of AKT phosphorylation and ROS may be a potential therapeutic approach targeting cancer stem cells. Further studies will be necessary to clarify in detail the molecular mechanism underlying enhancement of the stem cell phenotype of cancer cells under glucose depletion.

## Supplementary Materials

The following are available online at https://www.mdpi.com/2072-6694/11/12/1993/s1. Figure S1: The whole images of Western blotting, Figure S2: (a) Expression of the *BCL2* superfamily (*BCL2, MCL1* and *BAX*) in CCO1 and CCO2 cultured without glucose after treatment with the AKT phosphorylation inhibitor (AKTi), LY264002. (b) Expression of *mTOR* in CCO1 and CCO2 cultured without glucose after treatment with the AKT phosphorylation inhibitor (AKTi), LY264002, Table S1: GenBank accession number, primer sequence and melting temperature for each gene analyzed by quantitative RT-PCR are shown.

## Abbreviations

CCO	cholangiocarcinoma organoid
EGF	epidermal growth factor
ICC	intrahepatic cholangiocarcinoma
NAC	N-acetylcysteine
NOS	nitric oxide synthase
NOX	NADPH oxidase
PI3K	phosphatidylinositol 3-kinase
Rspo1	R-spondin 1
RT-PCR	quantitative reverse transcription-polymerase chain reaction
ROS	reactive oxygen species
SOD	superoxide dismutase

## References

[1] Weinhouse S., Warburg O., Burk D., Schade A.L. (1956). On Respiratory Impairment in Cancer Cells. Science.

[2] Potter V.R. (1958). The biochemical approach to the cancer problem. Fed. Proc..

[3] Hirayama A., Kami K., Sugimoto M., Sugawara M., Toki N., Onozuka H., Kinoshita T., Saito N., Ochiai A., Tomita M. (2009). Quantitative Metabolome Profiling of Colon and Stomach Cancer Microenvironment by Capillary Electrophoresis Time-of-Flight Mass Spectrometry. Cancer Res..

[4] Heddleston J.M., Li Z., Lathia J.D., Bao S., Hjelmeland A.B., Rich J.N. (2010). Hypoxia inducible factors in cancer stem cells. Br. J. Cancer.

[5] Ueki S., Murakami Y., Yamada S., Kimura M., Saito Y., Saito H. (2016). microRNA-mediated resistance to hypoglycemia in the HepG2 human hepatoma cell line. BMC Cancer.

[6] Razumilava N., Gores G.J. (2014). Cholangiocarcinoma. Lancet.

[7] Valle J., Wasan H., Palmer D.H., Cunningham D., Anthoney A., Maraveyas A., Madhusudan S., Iveson T., Hughes S., Pereira S.P. (2010). Cisplatin plus Gemcitabine versus Gemcitabine for Biliary Tract Cancer. N. Engl. J. Med..

[8] Rizvi S., Gores G.J. (2013). Pathogenesis, diagnosis, and management of cholangiocarcinoma. Gastroenterology.

[9] Razumilava N., Gores G.J. (2013). Classification, diagnosis, and management of cholangiocarcinoma. Clin. Gastroenterol. Hepatol..

[10] Sato T., Vries R.G., Snippert H.J., Van De Wetering M., Barker N., Stange D.E., Van Es J.H., Abo A., Kujala P., Peters P.J. (2009). Single Lgr5 stem cells build crypt-villus structures in vitro without a mesenchymal niche. Nature.

[11] Sato T., Stange D.E., Ferrante M., Vries R.G., Van Es J.H., Van den Brink S., Van Houdt W.J., Pronk A., Van Gorp J., Siersema P.D. (2011). Long-term expansion of epithelial organoids from human colon, adenoma, adenocarcinoma, and Barrett’s epithelium. Gastroenterology.

[12] Huch M., Dorrell C., Boj S.F., van Es J.H., Li V.S., van de Wetering M., Sato T., Hamer K., Sasaki N., Finegold M.J. (2013). In vitro expansion of single Lgr5+ liver stem cells induced by Wnt-driven regeneration. Nature.

[13] Huch M., Bonfanti P., Boj S.F., Sato T., Loomans C.J.M., Van De Wetering M., Sojoodi M., Li V.S.W., Schuijers J., Gracanin A. (2013). Unlimited in vitro expansion of adult bi-potent pancreas progenitors through the Lgr5/R-spondin axis. EMBO J..

[14] Saito Y., Nakaoka T., Sakai K., Muramatsu T., Toshimitsu K., Kimura M., Kanai T., Sato T., Saito H. (2016). Inhibition of DNA Methylation Suppresses Intestinal Tumor Organoids by Inducing an Anti-Viral Response. Sci. Rep..

[15] Nakaoka T., Saito Y., Shimamoto Y., Muramatsu T., Kimura M., Kanai Y., Saito H. (2017). Cluster microRNAs miR-194 and miR-215 suppress the tumorigenicity of intestinal tumor organoids. Cancer Sci..

[16] Saito Y., Nakaoka T., Muramatsu T., Ojima H., Sukeda A., Sugiyama Y., Uchida R., Furukawa R., Kitahara A., Sato T. (2018). Induction of differentiation of intrahepatic cholangiocarcinoma cells to functional hepatocytes using an organoid culture system. Sci. Rep..

[17] Yamada S., Takashina Y., Watanabe M., Nagamine R., Saito Y., Kamada N., Saito H. (2018). Bile acid metabolism regulated by the gut microbiota promotes non-alcoholic steatohepatitis-associated hepatocellular carcinoma in mice. Oncotarget.

[18] Yamada S., Kamada N., Amiya T., Nakamoto N., Nakaoka T., Kimura M., Saito Y., Ejima C., Kanai T., Saito H. (2017). Gut microbiota-mediated generation of saturated fatty acids elicits inflammation in the liver in murine high-fat diet-induced steatohepatitis. BMC Gastroenterol..

[19] Owada S., Shimoda Y., Tsuchihara K., Esumi H. (2013). Critical Role of H_2_O_2_ Generated by NOX4 during Cellular Response under Glucose Deprivation. PLoS ONE.

[20] Esumi H., Lu J., Kurashima Y., Hanaoka T. (2004). Antitumor activity of pyrvinium pamoate, 6-(dimethylamino)-2-[2-(2,5-dimethyl-1-phenyl-1H-pyrrol-3-yl)ethenyl]-1-methyl-qu inolinium pamoate salt, showing preferential cytotoxicity during glucose starvation. Cancer Sci..

[21] Izuishi K., Kato K., Ogura T., Kinoshita T., Esumi H. (2000). Remarkable tolerance of tumor cells to nutrient deprivation: Possible new biochemical target for cancer therapy. Cancer Res..

[22] Koshikawa N., Hayashi J., Nakagawara A., Takenaga K. (2009). Reactive oxygen species-generating mitochondrial DNA mutation up-regulates hypoxia-inducible factor-1alpha gene transcription via phosphatidylinositol 3-kinase-Akt/protein kinase C/histone deacetylase pathway. J. Biol. Chem..

[23] Du J., Xu R., Hu Z., Tian Y., Zhu Y., Gu L., Zhou L. (2011). PI3K and ERK-induced Rac1 activation mediates hypoxia-induced HIF-1alpha expression in MCF-7 breast cancer cells. PLoS ONE.

[24] Chen J. (2013). Potential value and limitation of dual inhibitors of PI3K and mTOR in the treatment of cancer. Curr. Cancer Drug Targets.

[25] Bu Z., Ji J. (2013). Therapeutic implications of mTOR inhibitors in the treatment of gastric cancer. Curr. Cancer Drug Targets.

[26] Cho D.C., Mier J.W. (2013). Dual inhibition of PI3-kinase and mTOR in renal cell carcinoma. Curr. Cancer Drug Targets.

[27] ElFiky A.A., Jiang Z. (2013). The PI3 kinase signaling pathway in prostate cancer. Curr. Cancer Drug Targets.

[28] Chen J., Shao R., Li F., Monteiro M., Liu J., Xu Z.P., Gu W. (2015). PI3K/Akt/mTOR pathway dual inhibitor BEZ235 suppresses the stemness of colon cancer stem cells. Clin. Exp. Pharmacol. Physiol..

[29] Holmström K.M., Finkel T. (2014). Cellular mechanisms and physiological consequences of redox-dependent signalling. Nat. Rev. Mol. Cell Biol..

[30] Liu L., Wise D.R., Diehl J.A., Simon M.C. (2008). Hypoxic reactive oxygen species regulate the integrated stress response and cell survival. J. Biol. Chem..

[31] Bensaad K., Cheung E.C., Vousden K.H. (2009). Modulation of intracellular ROS levels by TIGAR controls autophagy. EMBO J..

[32] Jiang F., Zhang Y., Dusting G.J. (2011). NADPH Oxidase-Mediated Redox Signaling: Roles in Cellular Stress Response, Stress Tolerance, and Tissue Repair. Pharmacol. Rev..

[33] Wu W.-S. (2006). The signaling mechanism of ROS in tumor progression. Cancer Metastasis Rev..

[34] Wang X., Martindale J.L., Liu Y., Holbrook N.J. (1998). The cellular response to oxidative stress: Influences of mitogen-activated protein kinase signalling pathways on cell survival. Biochem. J..

[35] Dalton T.P., Shertzer H.G., Puga A. (1999). Regulation of gene expression by reactive oxygen. Annu. Rev. Pharmacol. Toxicol..

[36] Griendling K.K., Harrison D.G. (1999). Dual role of reactive oxygen species in vascular growth. Circ. Res..

[37] Gào X., Schöttker B. (2017). Reduction–oxidation pathways involved in cancer development: A systematic review of literature reviews. Oncotarget.

[38] Nakanishi A., Wada Y., Kitagishi Y., Matsuda S. (2014). Link between PI3K/AKT/PTEN Pathway and NOX Proteinin Diseases. Aging Dis..

