# Supplementary material for: Glucose Depletion Enhances the Stem Cell Phenotype and Gemcitabine Resistance of Cholangiocarcinoma Organoids through AKT Phosphorylation and Reactive Oxygen Species
Source: Cancers (Basel). 2019 Dec 11;11(12):1993. doi: 10.3390/cancers11121993 (PMC6966500; doi:10.3390/cancers11121993)
Supplement: Supplementary file 1 [file cancers-11-01993-s001.pdf]

Supplementary Materials: Glucose depletion enhances the stem cell phenotype and gemcitabine resistance of cholangiocarcinoma organoids through AKT phosphorylation and reactive oxygen species

Nao Yoshikawa, Yoshimasa Saito, Hiroki Manabe, Toshiaki Nakaoka, Ryoei Uchida, Ryo Furukawa, Toshihide Muramatsu, Yuko Sugiyama, Masaki Kimura and Hidetsugu Saito

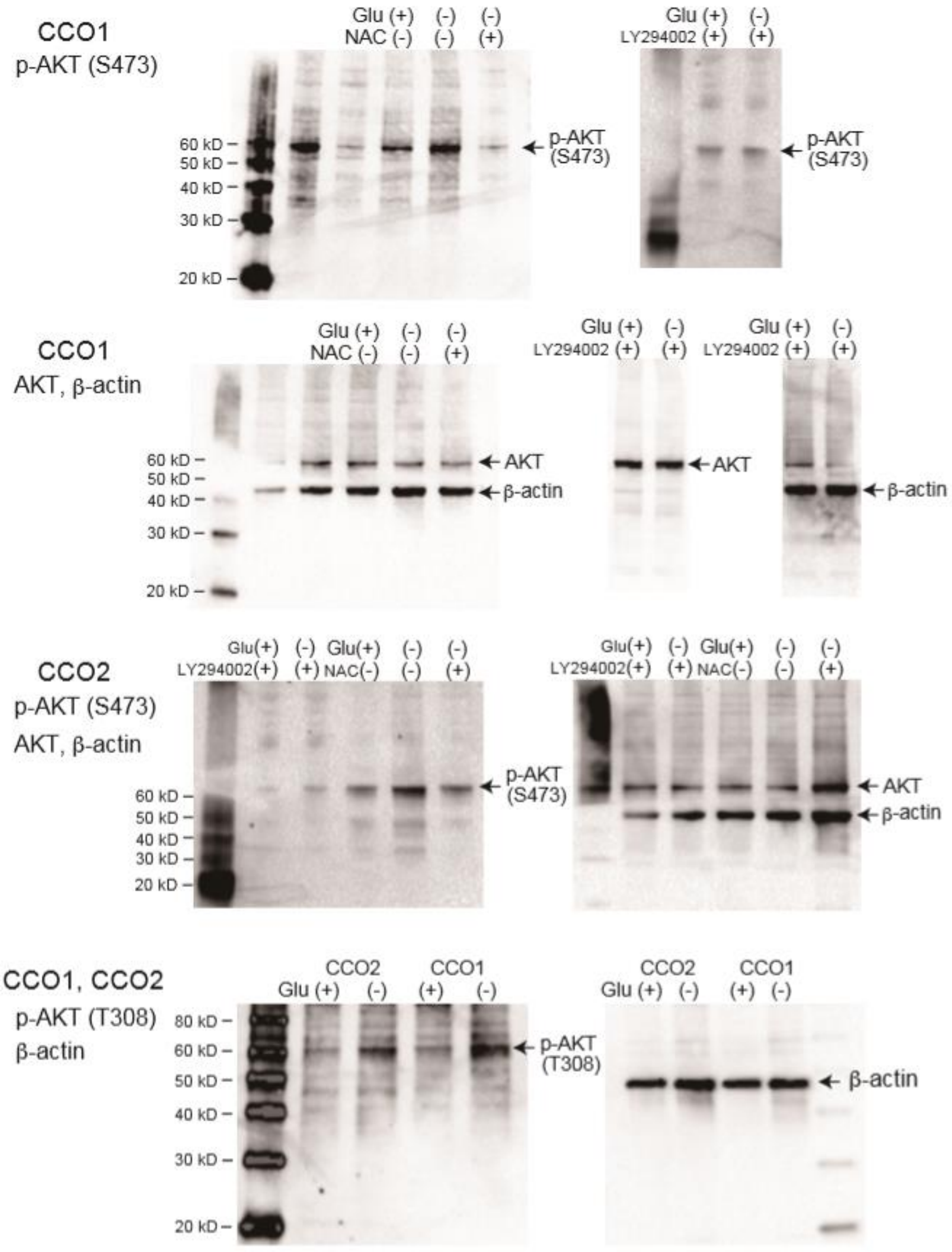

Figure S1. The whole images of Western blotting.

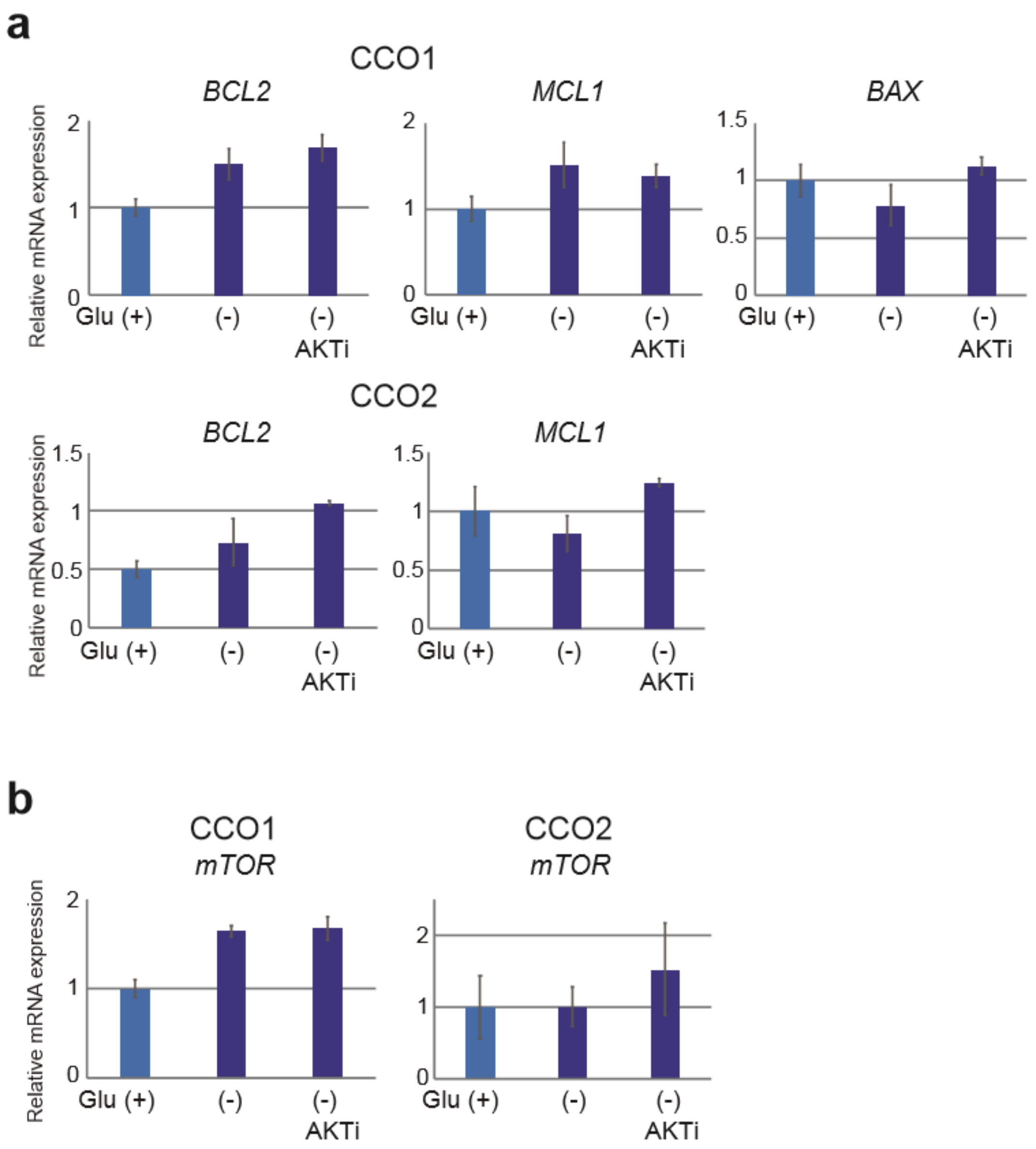

Figure S2. (a) Expression of the *BCL2* superfamily (*BCL2, MCL1 and BAX*) in CCO1 and CCO2 cultured without glucose after treatment with the AKT phosphorylation inhibitor (AKTi), LY264002. **(b)** Expression of *mTOR* in CCO1 and CCO2 cultured without glucose after treatment with the AKT phosphorylation inhibitor (AKTi), LY264002.

Table S1. GenBank accession number, primer sequence and melting temperature for each gene analyzed by quantitative RT-PCR are shown.

LGR5 (NM_003667)
Forward: 5'-TGCTGGCTGGTGTGGATGCG-3' (62℃)
Reverse: 5'-GCCAGCAGGGCACAGAGCAA-3' (62℃)
CD44 (NM_001001391)
Forward: 5'-CTGCCGCTTTGCAGGTGTA-3' (57℃)

Reverse: 5’-CATTGTGGGCAAGGTGCTATT-3’ (56℃)
EpCAM (NM_002354)
Forward: 5’-AGTTGGTGCACAAAATACTGTCAT-3’ (54℃)
Reverse: 5’-TCCCAAGTTTTGAGCCATTC-3’ (53℃)
NANOG (NM_024865)
Forward: 5’-TCTGGACACTGGCTGAATCC-3’ (57℃)
Reverse: 5’-TGACTGGATGGGCATCATGG-3’ (57℃)
OCT4 (NM_203289)
Forward: 5’-AGGTATTCAGCCAAACGACCA-3’ (56℃)
Reverse: 5’-GCACGAGGGTTTCTGCTTTG-3’ (57℃)
GAPDH (NM_002046)
Forward: 5’-CACCACCATGGAGAAG-3’ (52℃)
Reverse: 5’-GCTAAGCAGTTGGTGG-3’ (52℃)
